# Using an ACTIVE teaching format versus a standard lecture format for increasing resident interaction and knowledge achievement during noon conference: a prospective, controlled study

**DOI:** 10.1186/1472-6920-14-129

**Published:** 2014-07-01

**Authors:** Adam P Sawatsky, Kathryn Berlacher, Rosanne Granieri

**Affiliations:** 1 Division of General Internal Medicine, Mayo Clinic, 200 1st St SW, Rochester, MN, USA; 2 Division of Cardiology, University of Pittsburgh School of Medicine, Pittsburgh, PA, USA; 3 Division of General Internal Medicine, University of Pittsburgh School of Medicine, Pittsburgh, PA, USA

**Keywords:** Medical education, Graduate medical education, Conference, Active teaching, Lecture

## Abstract

**Background:**

The traditional lecture is used by many residency programs to fulfill the mandate for regular didactic sessions, despite limited evidence to demonstrate its effectiveness. Active teaching strategies have shown promise in improving medical knowledge but have been challenging to implement within the constraints of residency training. We developed and evaluated an innovative structured format for interactive teaching within the residency noon conference.

**Methods:**

We developed an ACTIVE teaching format structured around the following steps: assemble (A) into groups, convey (C) learning objectives, teach (T) background information, inquire (I) through cases and questions, verify (V) understanding, and explain (E) answer choices and educate on the learning points. We conducted a prospective, controlled study of the ACTIVE teaching format versus the standard lecture format, comparing resident satisfaction, immediate knowledge achievement and long-term knowledge retention. We qualitatively assessed participating faculty members’ perspectives on the faculty development efforts and the feasibility of teaching using the ACTIVE format.

**Results:**

Sixty-nine internal medicine residents participated in the study. Overall, there was an improvement in perceived engagement using the ACTIVE teaching format (4.78 vs. 3.80, P < 0.01), with no increase in stress or decrement in break time. There was an improvement in initial knowledge achievement with the ACTIVE teaching format (overall absolute score increase of 11%, P = 0.04) and a trend toward improvement in long-term knowledge retention. Faculty members felt adequately prepared to use the ACTIVE teaching format, and enjoyed teaching with the ACTIVE teaching format more than the standard lecture.

**Conclusions:**

A structured ACTIVE teaching format improved resident engagement and initial knowledge, and required minimal resources. The ACTIVE teaching format offers an exciting alternative to the standard lecture for resident noon conference and is easy to implement.

## Background

Within the Next Accreditation System, the Accreditation Council for Graduate Medical Education (ACGME) has established milestones within each of the core competencies [1]. To achieve the milestones for medical knowledge, the ACGME common program requirements mandate regularly scheduled didactic sessions, but allow flexibility for innovation in this area [2].

Traditional lecture within a noon conference setting has been the standard format for fulfilling the ACGME mandate [3]. There has been little data to support the lecture format, and most studies evaluating noon conference have shown no improvements in knowledge retention [4-6] or scores on national standardized exams [7-11]. We also know that residents desire an alternative to the traditional lecture that is more consistent with adult learning theory [12]. This lack of support has stimulated educators to develop new teaching strategies.

The choice of teaching method needs to be consistent with the learning goals [13]. The goal knowledge accumulation in residency is to equip residents for the “personal responsibility for the care of individual patients [2],” and therefore demands the ability to transfer that knowledge from the learning environment to actual patient care. Active learning can engage students in the learning process, and if questions are asked appropriately, can help learners move beyond knowledge acquisition to transfer of information to the patient environment [13,14].

Subsequently there have been several attempts to study other formats for teaching residents, including small groups, practice-based learning and team-based learning [15-17]. While there is excitement for the use of these formats, there are challenges in implementing them, including changes in the residency structure and increases in faculty development and oversight [17,18].

Because of the limitations of using the standard lecture and the challenges with other active teaching formats, we developed a new structured ACTIVE teaching format that would be easy to implement within resident noon conference. In this study, we sought to answer the following question: will the ACTIVE teaching format improve residents’ 1) satisfaction with learning; 2) immediate knowledge achievement; and 3) long-term knowledge retention. Additionally, we wanted to explore the faculty members’ responses to teaching using the ACTIVE format.

## Methods

### Setting

The structured ACTIVE teaching format was developed and studied at the University of Pittsburgh Internal Medicine Residency Program. As part of the current curriculum, a standard noon conference lecture series is given twice weekly, with topics that rotate throughout a three-year cycle. Lectures are given by the same faculty member twice at two separate locations, a university-based hospital and a VA hospital. Both locations are set up with tables in rows, with several chairs at each table.

This study was approved by the University of Pittsburgh Institutional Review Board.

### Structured format for ACTIVE teaching

In 2012, we conducted focus groups of residents and faculty to assess learning preferences, teaching perspectives within the noon conference, and perceived barriers to active teaching [12]. Using this data, we constructed the ACTIVE teaching format to integrate principles of adult learning, to address residents’ stated learning preferences and to facilitate faculty development in interactive teaching.

The ACTIVE teaching format facilitates small group interaction within a large group. It requires faculty members to focus on 3–5 learning points centered on cases and questions that allow for discussion (Table 1). This format outlines the following steps: learners assemble (**A)** into smaller groups (average 4 members). Before the lecture, chief residents distribute 8½” × 11” cards with the letters A-E printed on them to each group. These cards are printed on different colors or patterns for each group, and have been laminated for recurrent use. The facilitator conveys **(C)** the 3–5 learning points and then teaches **(T)** a limited amount of background material (3–5 minutes). The facilitator presents a case and inquires **(I)** of the group using a question about patient management. Each group discusses the question for 2–3 minutes and comes to consensus on their best answer. The facilitator verifies **(V)** their understanding by having each group hold up the card with their answer simultaneously and then debriefs the groups on rationale behind their answer choices. Then, the facilitator explains **(E)** the answer choices and educates the residents on the learning point. This process of inquiring, verifying, explaining and educating is then repeated for each learning point. The points are summarized at the conclusion of the conference. Facilitators were instructed that the whole conference was to last 45 minutes.

**Table 1:** Components of the ACTIVE teaching format

**ACTIVE teaching format**	
•	Assemble into groups
•	Convey learning objectives (3–5)
•	Teach background information
•	Inquire through cases and questions
•	Verify understanding through voting with cards and debriefing the groups
•	Explain answer choices and educate on the main learning points

We piloted the approach in June of 2012 to assess feasibility and congruence with resident learning preferences. The pilot conferences averaged 45 minutes, with each cycle of inquiring, verifying, explaining and educating lasting 8–10 minutes.

### Study design

Using a faculty champion, we recruited four faculty members from the division of cardiology to participate in the study. Faculty members were chosen based on their willingness to participate in the study, and no one who desired to participate was excluded. For each topic, the faculty member gave the intervention conference using the ACTIVE format at the university-based hospital and the control conference using the standard lecture format at the VA hospital.

### Faculty development

The study investigators gave a one-hour presentation on the new format to participating faculty and showed a video clip example. Faculty members chose their topics from the residency curriculum and were asked to develop or update a standard lecture on that topic. They were then to transform their lecture into the ACTIVE format. The principal investigator (AS) met with each faculty member for one hour to provide additional assistance, centered on developing focused learning points and case-based questions. All follow-up outside of these sessions was minimal and was done over e-mail.

### Participants

We invited all categorical internal medicine, medicine-pediatric, preliminary and transitional year residents to participate in the study. Residents had been randomly assigned to their hospital rotation and location at the beginning of the academic year. The study took place during the required noon conference series. Participants were chosen at random to win a 25 dollar gift card for their participation in the study, with an additional 25 dollar incentive for those participants who completed the entire study.

### Assessments

We assessed learner satisfaction, initial knowledge achievement and long-term knowledge retention. To assess learner satisfaction, we administered a survey evaluating residents’ perceived gain in knowledge, appropriateness of content, clarity of learning points, relevance of learning points, engagement in learning, and enthusiasm of the lecturer. To evaluate the negative effects of the format change, we surveyed the stressfulness of the conference, the ability to take a break in their day and to eat lunch. The survey was given to each participant immediately following each lecture, and again 4–6 weeks after the lecture series ended. Each survey item was scored on a Likert scale from 1 to 5 (strongly disagree to strongly agree).

To assess initial knowledge achievement, we distributed 5 multiple-choice questions based on topics covered in the conference. These questions were constructed similarly to those in the American Board of Internal Medicine certification exam. To assess long-term knowledge retention, we distributed 20 multiple-choice questions (the same 5 questions distributed at the end of each lectures) 4–6 weeks after the end of the lecture series.

To assess faculty satisfaction with using the ACTIVE format, we conducted semi-structured interviews to explore their comfort with preparation and delivery of the format. The interview guide was developed by the research team to reflect the important aspects of preparation and delivery of the ACTIVE format. The interviews were recorded. The recorded interviews were analyzed using thematic analysis. Major themes were identified by one of the authors (AS) and reviewed with the research team, including one of the facilitators, for accuracy.

Because of non-normal distributions, we compared resident satisfaction and initial knowledge achievement scores using the Wilcoxon rank sum test. We compared long-term knowledge retention using a two-sided t-test.

## Results

### Participants

Of the 144 categorical internal medicine, medicine-pediatric, preliminary and transitional year residents, 80 (56%) participated in the study; 54 residents (38%) participated in the initial assessment (Table 2). Not all residents attended each session, so the number of participants in each session is less than the total number of participants. The 4 participating faculty members ranged from third year cardiology fellow to senior faculty with more than 20 years of teaching experience. They presented conferences on four topics: pericarditis, ECG reading, congestive heart failure and endocarditis.

**Table 2:** Demographics of participating residents

	**Conference format**	
	**ACTIVE teaching format (n = 45)**	**Standard lecture (n = 9)**
Gender		
Male	25 (56%)	4 (44%)
Post-Graduate Year (PGY)		
PGY-1	21 (47%)	7 (78%)
PGY-2	9 (20%)	0
PGY-3	15 (33%)	2 (22%)
Program		
Categorical	35 (78%)	6 (67%)
Preliminary	6 (13%)	2 (22%)
Transitional	4 (9%)	1 (11%)
Average Sessions Attended	1.8	2.6

### Resident satisfaction

For each individual topic, there was no difference between the ACTIVE teaching format and the standard lectures in residents’ perceived knowledge gain, content appropriateness, or lecturer enthusiasm. For the ECG reading conference, the ACTIVE format demonstrated improvements in learning point clarity (4.84 vs. 4.00, p < 0.01) and relevance (4.94 vs. 4.5, p < 0.01). There was a trend toward improved engagement using the ACTIVE format in all individual conferences. There were no differences in stressfulness or ability to take a break from clinical workload and eat lunch for any of the individual topics.

There was a statistically significant difference in overall engagement between the ACTIVE conference series and the standard lecture series (4.78 vs. 3.80, P < 0.01), with no increase in stress or decrement in break time.

### Immediate knowledge achievement

Residents who attended the ACTIVE conference series scored higher on the immediate knowledge questions when compared to residents who attended the standard lecture series, with an absolute improvement in score of 11% (Table 3).

**Table 3:** Immediate knowledge achievement with the ACTIVE teaching format versus the standard lecture format

**Immediate knowledge test**					
	**Active teaching format**		**Standard lecture format**		***P *** **value (Rank Sum Test)**
	**Mean**	**n**	**Mean**	**n**	
**Session 1 (Pericarditis)**	86%	23	72%	5	0.05
**Session 2 (ECG)**	70%	29	65%	4	0.50
**Session 3 (CHF)**	82%	29	60%	7	0.04
**Session 4 (Endocarditis)**	75%	21	73%	6	0.67
Overall	80%	45	69%	9	0.04

### Long-term knowledge retention

Because of poor response to the retention knowledge test from the control group, we gave the same assessment to residents who did not attend any lecture in the study month. We compared the scores for those who attended ACTIVE conference series (n = 21) to non-attenders (n = 26), finding a trend toward improvement in the ACTIVE conference group (71% vs. 65%, p = 0.15). Compared to the non-attenders, the ACTIVE conference group had more categorical residents (90% vs. 73%) and more residents who had a cardiology rotation in the previous 6 months (62% vs. 46%). The ACTIVE conference group had less upper-level residents (62% vs. 81%) and less residents interested in cardiology (5% vs. 19%).

### Faculty satisfaction

Prior to participation in the study, faculty had two main reservations. They were concerned about learning the new ACTIVE teaching format, and asked to see a visual example before using the format. They also did not want to spend more time on the ACTIVE teaching format than they would usually spend preparing their standard lecture.

Faculty discussed the processes of preparing and delivering each of the conferences. Faculty members felt comfortable with the preparation in the ACTIVE teaching format. They took between 5–15 hours to prepare the talk, not significantly different than the time it would usually take them to prepare a standard lecture. The biggest challenges were developing good questions and limiting the amount of included information. Creating good questions that would generate discussion took some creativity, and they felt this is where the most assistance was needed. One faculty member reflected on this process, and felt like it caused her to put herself in the “learners’ mindset.” All faculty members felt that the amount of faculty development time was adequate, stating that the one-on-one sessions were the most beneficial for “bouncing ideas” off an expert and receiving feedback about the format. The video example used for faculty development was also useful.

All faculty members agreed that using the ACTIVE teaching format was enjoyable, and several faculty members felt more comfortable with this format compared to the standard lecture. They sensed that the residents were more engaged in the learning process, evidenced by eye contact, body language and increase in discussion. They appreciated the immediate feedback from learners using the cards, and the built in repetition and summarization. Faculty discussed the challenges of the group discussion; they felt they had limited ability to monitor the quality of group discussions. While there was more interaction among the learners, faculty members expressed some difficulty with interaction between the teacher and the learners.

The faculty agreed that they could present in this format again, and many expressed that it should be a requirement for the residency, if given adequate support from the program. Faculty members suggested areas for future exploration, including the appropriate size and composition of each small group, guidelines for faculty interaction and optimal topics for the format.

## Discussion

We designed a structured format for interactive teaching of residents during noon conference and we examined its impact on resident satisfaction, knowledge acquisition and long-term knowledge retention. This simple, structured intervention improved residents’ initial knowledge achievement and satisfaction with engagement. The format did not cause an increase in stress or a decrement in the ability to take a break in the day and eat lunch, important outcomes to residents [12]. The time needed for faculty development was reasonable and faculty participants felt they could continue to implement this format with little additional assistance. The ACTIVE teaching format shows promise as a method for teaching residents within the noon conference, as it allows for increased engagement without major structural changes to the residency and preserves other resident-perceived benefits of noon conference.

Other residency programs have attempted to increase engagement in their conferences through the use of team-based learning [17,18]. While our format may share some of the principles of team-based learning, there are many key differences. First, the ACTIVE teaching format does not require residents to prepare anything prior to attending conference. Second, the questions asked in the ACTIVE structure are not tied to a grade, unlike the readiness assurance tests in team-based learning. This allows the facilitator to ask a question that may have more than one right answer, which generates more fruitful discussion. Third, we did not structure the composition of the groups. We felt that this would be difficult with the variability of resident attendance at conference. Fourth, the ACTIVE structure can be completed within the allotted time for noon conference. Most team-based learning models take more than one hour, and have necessitated moving to an academic half-day [18]. These differences enhance the feasibility of using the ACTIVE teaching format in residency education.

Another interesting component of the ACTIVE teaching format is the use of the card system for answering questions instead of an audience response system (ARS). The ARS is a safe way to engage individual learners anonymously within large groups settings [19,20]. When we discussed the use of ARS with our residents, many felt that it was not adequate at engaging them in lecture [12]. A majority of the literature that studied student and resident satisfaction with ARS was done in the setting of newly implemented ARS [21-23], and our residency program has been using this system for several years, which may lead to decrease satisfaction over time. Also, there is literature to suggest that it is not the ARS, but the use of questions in lectures that leads to improvements in knowledge [24]. We purposefully used large cards because they provided accountability for the learners to their answers and allowed them to compare and contrast their answers to their peers. Additionally, it gave better visual feedback to the facilitator, who could debrief specific groups and tailor comments to the specific learning needs of the groups. To address safety of the learning environment, residents were able to discuss their answers with other residents in small groups before committing within the larger group. We demonstrated that this approach did not increase the learners’ sense of stress over regular lecture.

There were a few challenges to implementing this ACTIVE format. Many faculty members raised the concern that this format reduces the amount of information delivered to the learners during the conference. We know from previous studies that the lecture format did improve resident knowledge retention [4-11], and we hypothesized that by decreasing the amount of information and increasing learner participation, we would improve overall knowledge retention. Unfortunately, we did not demonstrate an improvement in retention in the sample that we observed. Many faculty members were also concerned about loss of control over the learning environment when using active learning in a large group setting. We anticipated that by providing specific structure to the active learning, the faculty would maintain a sense of control. Faculty members were comfortable using the ACTIVE format, but still were concerned because they could not monitor the quality of discussion in each individual small group. Finally, the faculty members volunteered to participate in the study and were willing to teach using a new format. Not all faculty members may be willing to use this format. While the response to the ACTIVE format was positive, implementation within the residency program would require faculty and resident buy-in.

We acknowledge several limitations of this study. This study was performed at a single large academic institution. This may limit the generalizability, but the format can be adapted to fit other residency structures easily. This is a low cost intervention that does not require special technology. While we studied a limited number of topics, we believe the ACTIVE format can be applied to any topic in medicine; we did study topics that included both management topics (pericarditis, congestive heart failure and endocarditis) as well as skill acquisition (ECG reading). Another limitation is that we used the same faculty for the control and intervention and the faculty delivered the control lecture after receiving faculty development. This could have affected their performance on the control lecture. However, we felt the use of the same lecturer controlled for confounders, and that any spill-over effect would only bias towards no difference between the intervention and control groups. We did not do pre-test assessments of the learners, but the semi-random nature of the assignment should control for any confounders in previous achievement. While there were differences between baseline characteristics of the ACTIVE learning group and non-attenders, there was a balance of characteristics that could drive findings in either direction. We also did not want to burden the participants with additional testing. Our study included a small sample of participants, which reflects the challenge of studying a classroom environment in residency.

## Conclusions

Implementing a structured, active format for teaching residents in the noon conference setting showed a beneficial impact on resident engagement and initial knowledge gained, with a trend toward improved knowledge retention. Faculty preferred using the format to their standard lectures, and highlighted the advantages of resident engagement, immediate learner feedback, repetition and summarization. The structured format was easy to implement, requiring minimal faculty development time or residency program structure change. It can be easily implemented in diverse settings and adapted for use in different specialties. The ACTIVE format provides an additional teaching method for fulfilling ACGME requirements for regular didactic sessions.

## Abbreviations

ACGME: Accreditation council for graduate medical education; ARS: Audience response system.

## Competing interests

The authors declare that they have no competing interests.

## Authors’ contributions

AS developed the ACTIVE teaching format and participated in the research design, data acquisition, analysis and interpretation, and drafted the manuscript. KB participated in the research design, data acquisition and interpretation, and critical review of the manuscript. RG participated in the research design, data interpretation, and critical review of the manuscript. All authors read and approved the final manuscript.

## Pre-publication history

The pre-publication history for this paper can be accessed here:

http://www.biomedcentral.com/1472-6920/14/129/prepub
